# Lysogeny with Shiga Toxin 2-Encoding Bacteriophages Represses Type III Secretion in Enterohemorrhagic *Escherichia coli*

**DOI:** 10.1371/journal.ppat.1002672

**Published:** 2012-05-17

**Authors:** Xuefang Xu, Sean P. McAteer, Jai J. Tree, Darren J. Shaw, Eliza B. K. Wolfson, Scott A. Beatson, Andrew J. Roe, Lesley J. Allison, Margo E. Chase-Topping, Arvind Mahajan, Rosangela Tozzoli, Mark E. J. Woolhouse, Stefano Morabito, David L. Gally

**Affiliations:** 1 Immunity and Infection Division, The Roslin Institute and R(D)SVS, The University of Edinburgh, Easter Bush, Midlothian, United Kingdom; 2 Veterinary Clinical Sciences, The Roslin Institute and R(D)SVS, The University of Edinburgh, Easter Bush, Midlothian, United Kingdom; 3 School of Chemistry and Molecular Biosciences and Australian Infectious Diseases Research Centre, The University of Queensland, Brisbane, Australia; 4 Institute of Infection, Immunity and Inflammation, College of Medical, Veterinary and Life Sciences, Glasgow Biomedical Research Centre, Glasgow, United Kingdom; 5 Scottish E. coli VTEC Reference Laboratory, Department of Laboratory Medicine, Royal Infirmary of Edinburgh, Edinburgh, United Kingdom; 6 Centre for Immunity, Infection & Evolution, Ashworth Laboratories, University of Edinburgh, Edinburgh, United Kingdom; 7 European Union Reference Laboratory for Escherichia coli, Istituto Superiore di Sanitá, Dipartimento di Sanitá Pubblica Veterinaria e Sicurezza Alimentare, Rome, Italy; Northwestern University Feinberg School of Medicine, United States of America

## Abstract

Lytic or lysogenic infections by bacteriophages drive the evolution of enteric bacteria. Enterohemorrhagic *Escherichia coli* (EHEC) have recently emerged as a significant zoonotic infection of humans with the main serotypes carried by ruminants. Typical EHEC strains are defined by the expression of a type III secretion (T3S) system, the production of Shiga toxins (Stx) and association with specific clinical symptoms. The genes for Stx are present on lambdoid bacteriophages integrated into the *E. coli* genome. Phage type (PT) 21/28 is the most prevalent strain type linked with human EHEC infections in the United Kingdom and is more likely to be associated with cattle shedding high levels of the organism than PT32 strains. In this study we have demonstrated that the majority (90%) of PT 21/28 strains contain both Stx2 and Stx2c phages, irrespective of source. This is in contrast to PT 32 strains for which only a minority of strains contain both Stx2 and 2c phages (28%). PT21/28 strains had a lower median level of T3S compared to PT32 strains and so the relationship between Stx phage lysogeny and T3S was investigated. Deletion of Stx2 phages from EHEC strains increased the level of T3S whereas lysogeny decreased T3S. This regulation was confirmed in an *E. coli* K12 background transduced with a marked Stx2 phage followed by measurement of a T3S reporter controlled by induced levels of the LEE-encoded regulator (Ler). The presence of an integrated Stx2 phage was shown to repress Ler induction of LEE1 and this regulation involved the CII phage regulator. This repression could be relieved by ectopic expression of a cognate CI regulator. A model is proposed in which Stx2-encoding bacteriophages regulate T3S to co-ordinate epithelial cell colonisation that is promoted by Stx and secreted effector proteins.

## Author Summary

Many significant infectious diseases that impact human health evolve in animal hosts. Our work focuses on infections caused by strains of enterohemorrhagic *Escherichia coli* (EHEC) that cause bloody diarrhoea and life threatening kidney and brain damage in humans as an incidental host, while ruminants are a reservoir host. EHEC strains are infected with bacteriophages that can integrate their genetic material into the bacterial chromosome. This includes genes for the production of Shiga toxins (Stx) that are responsible for the severe pathology in humans. It has been demonstrated that certain EHEC strains are more likely to be associated with human disease and ‘supershedding’ animals. The current study has shown that these EHEC strains are more likely to contain two related Stx bacteriophages, rather than one, and that the intercalating bacteriophages take control of the bacterial type III secretion system that is essential for ruminant colonization. We propose that this regulation favours co-acquisition of other genetic regions that encode type III-secreted proteins and regulators that can overcome this control. This finding helps our understanding of EHEC strain evolution and indicates that selection of more toxic strains may be occurring in the ruminant host with important implications for human health.

## Introduction

Bacteria such as *Escherichia coli* that colonise the mammalian gastrointestinal tract are exposed to high levels of bacterial viruses, bacteriophages [1]. Many bacteriophages inject their genetic material and use the bacterial host simply to produce more phage in a lytic cycle. Other, temperate phages, can insert their genetic material into the bacterial genome [1]–[4]. In this lysogenic state the phage genome is amplified along with the dividing bacteria [4]. Lysogenic phages have evolved to initiate their replication and escape from the bacterial host by using the stress or SOS response of the bacterium [5]. The integration of phage genomes and their subsequent degeneration is important in the evolution of many bacterial genera, for example *Salmonella*, *Staphylococcus* and *Escherichia* [6], [7].

Typical enterohemorrhagic *E. coli* (EHEC) are defined by the presence of Shiga toxin (Stx)-encoding lambdoid-like bacteriophages in the chromosome and the ability to form attaching and effacing lesions on epithelial cells of the gastrointestinal tract due to the activity of a type III secretion (T3S) system [8]–[11]. This secretion system is expressed from the locus of enterocyte effacement (LEE) and is controlled by multiple regulatory inputs, many through the LEE-encoded regulator (Ler). The T3S system translocates a cocktail of effector proteins into eukaryotic cells, many of which were introduced originally into the EHEC chromosome encoded on prophages along with regulators that co-ordinate effector expression with the T3S and may allow these non-LEE encoded effectors to compete for translocation [12]–[15]. While many of these prophages are considered cryptic their presence in strains with active prophages generates the constant possibility of new bacteriophage generation and transduction [16].

The Stx bacteriophages also introduce further variation into the strains, including the expression of different variants of Stx [17]. All Stx types belong to a family of A_1_B_5_ exotoxins comprising of a single A subunit that is non-covalently associated with pentameric B subunits that are responsible for toxin binding to its receptor, the glycosphingolipid Gb3 (globotriaosylceramide; CD77) [17]–[19]. The A subunit of Stx is an N-glycosidase which cleaves the N-glycosidic bond of a specific adenine residue of the 28S rRNA in the 60S ribosomal subunit inhibiting protein synthesis [17], [20]. Severe EHEC infections in humans are characterised by bloody diarrhoea and capillary damage in the kidneys and brain as a result of Stx activity with potential fatal consequences or long-term morbidity [1], [21], [22].

Humans are generally considered to be an incidental host for the majority of EHEC strains and it is apparent that ruminants [23], [24], in particular cattle are the main reservoir for the EHEC serotypes associated with human infection, in particular EHEC O157:H7 and EHEC O26:H11 [25], [26]. The pathogenesis of the organism therefore needs to be considered in the context of the ruminant host in terms of selective factors that drive its evolution. In contrast to the situation in humans, there is little evidence for pathology associated with EHEC infection in mature, immuno-competent cattle or other reservoir hosts which typically carry EHEC asymptomatically [27], [28]. This is related, in part, to differences in Gb3 receptor distribution in cattle versus humans and differences in how internalised toxin is trafficked in the cell [29]–[31]. If phage insertion can confer an advantage to the bacterium then this will also increase the survival chances of the integrated phage DNA. Studies on Shiga toxin activity have provided some insight into its possible benefits for EHEC in the ruminant host, even though a subset of the bacteria must lyse to release the toxin. Advantages for colonisation include the redistribution of nucleolin to the epithelial cell surface where it aids bacterial attachment via interaction with the bacterial outer membrane protein intimin [32]. Stx has also been shown to have immuno-modulatory functions, including the suppression of inflammatory responses and repression of B cell/T cell proliferation [33], [34], which could complement T3S effector repression of innate responses [35]–[39].

Epidemiological studies have shown that particular strain types emerge that are more likely to be associated with human disease. In the USA, Clade 8 is associated with more serious human disease [40] and in Europe particular phage types are more commonly associated with human infections [41]. In a previous study, Chase-Topping *et al* [42] investigated fecal pat samples from 481 farms throughout Scotland for the presence of *E. coli* O157:H7. Three main phage types were identified, 21/28 (46%), 32 (19%), and 8 (12%). Previous research had shown that cattle colonized at the terminal rectum can shed bacteria at high levels leading to the concept of ‘super-shedders’ [43], [44]. Low or high-level shedding was defined by whether the *E. coli* O157 counts were below or above 10^3^ CFU g^−1^ feces respectively. PT21/28 strains were more likely to be associated with high shedding and PT32 with low-level shedding samples. PT21/28 strains are the predominant type associated with human infection in the UK [44]. The initial aim of this study was to identify genetic differences between PT21/28 and PT32 strains. The research indicated that Stx bacteriophage carriage was different between the two as was the regulation of T3S. This led us to determine the impact of Stx phage lysogeny on T3S regulation in EHEC with the conclusion that Stx2-encoding bacteriophages usurp control of this essential colonization factor.

## Results

### Comparative genomic hybridization of PT 21/28 and PT 32 strains

**Figure 1:**
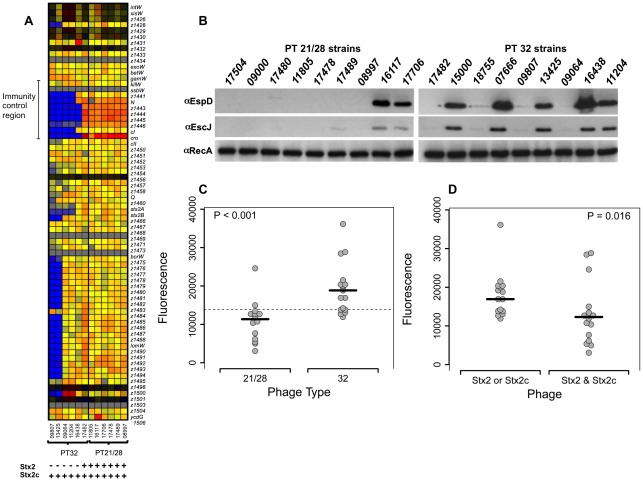
Comparison of EHEC O157 PT 21/28 and PT32 strains. (A) Comparative genome hybridisation of six PT21/28 and six PT32 strains. From left to right the PT21/28 strains are: 09807, 13425, 09064, 11204, 16438 & 17482; the PT32 strains are: 11805, 16117, 177706, 17478, 17489 & 08997 (table S1). The heat map shown indicates relative hybridisation levels of the defined strains to the Stx2 phage genome and flanking gene sequences from the O157:H7 Sakai strain which does not contain a Stx2c prophage. Red through to orange indicates a positive hybridization signal, with yellow a weakening signal through to blue colouration indicative of poor hybridization. All the PT21/28 strains show good relative hybridization to the sequences especially the *gam*-*cII* immunity region that is indicated. This is not the case for the PT32 strains with only 1 showing positive hybridisation over this same region, indicating that the remaining 5 strains are unlikely to contain the Stx2 phage. This was confirmed for the strains using a PCR to detect Stx2 and Stx2c phage control regions (Materials and Methods), the results of which are shown under the heat map lanes. The Stx phage distribution was then determined by PCR for 60 strains (table S1), please see Results section. (B) Western blot analysis of a subset of PT21/28 and PT32 strains as defined (table S1). EspD was detected from bacterial supernatants and EscJ and RecA from whole cell samples prepared as described in Materials and Methods. (C) Relative fluorescence levels from a LEE1-GFP reporter construct transformed into 14 PT21/28 and 16 PT32 strains with levels measured at OD_600_ = 1. The strains with mean values are defined in table S1. The median level of fluorescence from the PT32 strains is significantly higher than for the PT21/28 (p<0.001), the variability in expression levels correlates with the Western blotting data in part B. Also included is the overall median level (horizontal dashed line) (D) LEE1-GFP expression levels compared between strains containing either one or both Stx2 or Stx2c prophages. Strains containing both Stx2 phages have a significantly reduced (P = 0.016) median level of LEE1 expression compared with strains containing only one type of Stx2 prophage as determined at OD_600_ = 1 cultured in MEM-HEPES medium.

In order to investigate genomic differences between Phage types 21/28 and 32, twelve strains (six of each type) were analysed by comparative genomic hybridization (CGH) as detailed in Materials and Methods. These strains were chosen to include strains originating from fecal pats with both high and low bacterial counts (table S1). While multiple differences in gene content were identified between the phage types (GEO accession number GSE28838) the most consistent difference was in hybridization to the Stx2 bacteriophage immunity region. Only one PT32 strain from the six analyzed showed hybridization to this immunity region in contrast to all six PT21/28 strains that were investigated (figure 1A). The array design is based on the *E. coli* K-12, EDL933 and Sakai sequences (figure 1A). While EDL933 and Sakai contain both Stx1- and Stx2-encoding prophages they do not contain an Stx2c-encoding prophage. Previous characterization of the strain collection by the Scottish *E. coli* reference laboratory had determined that they all contained at least one type of Stx2 phage (table S1), so to investigate whether Stx2 vs Stx2c prophages could account for the hybridization differences, genomic DNA preparations from strains (n = 62) were analyzed for these prophages by PCR (Materials and Methods). While the majority of all the strains analyzed contained the Stx2c prophage (87%), the main difference was in the distribution of Stx2 prophages. Overall, examining both human and bovine-derived strains (table S1), 27/30 PT21/28 strains contained both an Stx2 and Stx2c-encoding prophage, compared with 9/32 of the PT32 strains analysed (P<0.01). This difference was still significant when examining bovine strains only (P<0.01) (table S1). It was interesting to note however that PT32 strains from humans were more likely (p = 0.01) to contain a Stx2-encoding prophage than the PT32 bovine isolates (75% versus 25%), while the majority of 21/28 strains from either source contained a Stx2-encoding prophage (table S1). So overall, PT21/28 strains, which are the main human disease associated phage type in the United Kingdom, generally harbor both Stx2 and Stx2c phages whereas PT32 strains were more likely to contain only a single Stx2 or Stx2c-encoding prophage, but with some further selection for Stx2-encoding prophages in the human-derived isolates.

### T3S regulation in PT 21/28 and PT 32 strains

The secretion profiles of thirty strains of each phage type were analysed, including western blotting for the translocon protein EspD (a subset of strains is shown in figure 1B). From these initial profiles it was evident that more PT32 strains were secreting higher levels of EspD. The strains also produced variable levels of EscJ, with higher levels of the T3S apparatus protein EscJ correlating with higher levels of EspD secretion (figure 1B). In order to quantify any differences in the expression of the T3S system in the two phage types, a subset of the strains (n = 31) were transformed with a LEE1::GFP reporter construct that measures transcription initiation of the first LEE operon including *ler* that encodes a key regulator of the T3S system [45]. Fluorescence levels were determined for the different strains during growth curves in MEM-Hepes as defined in the Materials and Methods. For statistical analyses, fluorescence values were determined from multiple experiments for OD_600_ = 1.0 (table S1). PT32 strains had a significantly higher median level of expression compared to the PT21/28 strains (p<0.001, figure 1C). As an alternative way of analysing potential differences in LEE1 expression between the PTs, the number of strains exhibiting expression levels above or below the median value of all the readings (13,846) was compared. On this basis, PT32 strains were more likely to express the LEE1 fusion at higher levels than the PT21/28 strains (p<0.01), consistent with the secretion profiles and Western blotting data. There was no association between T3S expression and single point shedding levels of the strain obtained from the fecal pat at the time of sampling (table S1).

Based on the observation that PT 21/28 was more likely to contain two types of Stx2-encoding prophage (Stx2 and Stx2c) and generally demonstrated lower levels of T3S, LEE1 expression levels were compared with the presence of Stx2 and Stx2c prophages. Strains containing both Stx2 and Stx2c prophages were significantly more likely (*p*<0.05) to have lower LEE1 expression levels than strains containing just one Stx2 prophage (figure 1D). The differences in T3S and LEE1 expression appeared consistent with differences in Stx2 and Stx2c prophage content, with more repression of T3S evident in strains containing both Stx2 and Stx2c prophages. As only one of the PT21/28 and PT32 strains analysed contained a Stx1 phage (table S1) it was not possible from this data to examine any impact of Stx1 prophage integration on T3S.

### Comparison of T3S expression in the presence and absence of Stx phages

**Figure 2:**
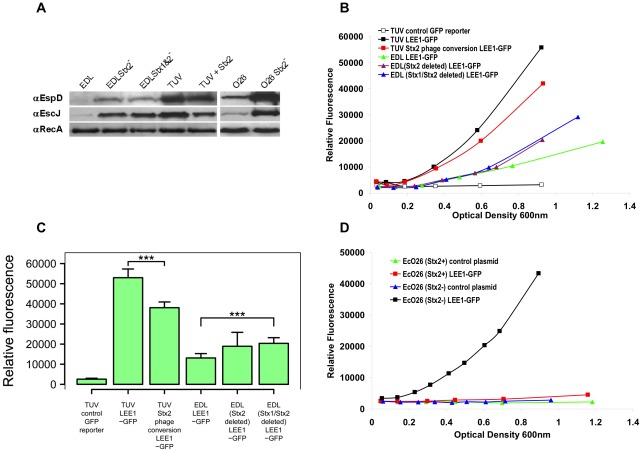
Analysis of T3S and LEE1 expression in *E. coli* with and without integrated Stx prophages. (A) Western blot analysis of paired *E. coli* O157 and O26 strains. EspD was detected from bacterial supernatants and EscJ and RecA from whole cell samples prepared as described in Materials and Methods. EDL is the sequenced EDL933 strain and this was originally compared with a published strain TUV93-0 (lane 4) that has lost both Stx1- and Stx2-encoding prophages. This strain demonstrates higher secretion levels and EscJ expression. The Stx2-encoding prophage and the Stx1-encoding prophage were deleted from EDL933 (lanes 2 and 3 respectively) leading to increased T3S. A marked Stx2 phage [67] was conjugated into TUV93-0 expressing a cloned *cI* from Sakai Sp5 (lane 5) and this resulted in a reduction in T3S and EscJ expression. The final two lanes contain samples from a published isogenic pair of EHEC O26 strains [47] from which one has lost the Stx2 phage leading to a marked increase in T3S. (B–C) To quantify differences in T3S expression, the same strain sets as in part A, were transformed with a LEE1-GFP construct and fluorescence measured throughout the growth curve. This was repeated three times with one experiment shown in (B). A minimum of three values were determined from the expression curves for OD_600_ = 0.9 and plotted in (C) as mean and 95% confidence intervals. *** : *p*<0.001 for the TUV strains and *p* = 0.001 for the EDL strains compared. (D) The *E. coli* O26 pair of strains blotted in (A) (table 1) were transformed with the LEE1-GFP expression construct or a control plasmid and population fluorescence levels measured through the growth curve. Taken together the data demonstrates that lysogeny with an Stx2-encoding phage represses T3S expression.

In order to investigate whether the expression of the T3S system is repressed by the presence of Stx prophages, secretion profiles and LEE1 expression levels in EHEC strains, with and without integrated Stx prophages, were analyzed. The sequenced isolate EDL933 was compared with a published Stx1 and Stx2 prophage-cured derivative TUV93-0 (figure 2A). Higher levels of EspD secretion were detected in bacterial supernatants of TUV93-0 compared with EDL933 (figure 2A). This correlated with higher levels of the apparatus protein, EscJ, in the whole cell fractions compared with RecA as a control (figure 2A). Again, to quantify this difference, the LEE1 promoter fusion was introduced into both strains. The expression in TUV93-0 was higher than EDL933 throughout the growth curve with a significance difference for values determined at OD_600_ = 0.9 (figure 2B–C). As both the EDL933 and TUV93-0 strains used have been cultured in a number of different laboratories and EDL933 was also subject to genetic manipulation during which phage deletion occurred [46], there may be other genetic changes, in addition to the absence of the Stx phages, that may account for the increased level of T3S in TUV93-0. To investigate this, two approaches were taken. In the first, a Stx2 bacteriophage (Sp5) marked with a kanamycin resistance cassette from a derivative *E. coli* O157 Sakai strain (Sakai *stx2A::kan*, table 1) was moved in the TUV93-0 background. Attempts to transduce this marked Sp5 phage into this background were not successful. However, conjugation of the prophage into TUV93-0 worked in the presence of a cloned and induced copy of the Sp5 *cI* (pBAD-CI, table S2) to restrict prophage induction. EspD secretion level, EscJ expression and LEE1-GFP levels were then determined in the absence of arabinose-induced *cI* induction (figure 2A–B). The conjugation led to the repression of T3S but not to the level demonstrated for EDL933. This repression was confirmed by analysis of both *ler* and *espD* transcript levels in the TUV strain pair, with TUV93-0(Sp5) showing a significant reduction in these T3S-associated transcripts in comparison to the parent strain (TUV93-0), while there was no significant change in *gapA* transcript levels between the two strains (figure S1). As a second approach, another series of strains were generated from EDL933 in which the Stx2-encoding prophage and then the Stx1-encoding prophage were deleted by allelic exchange to generate another Stx prophage negative derivative of EDL933. This strain exhibited increased levels of T3S and LEE1 expression by comparison with the isogenic EDL933 parent, but at lower levels than shown for TUV93-0 (figure 2A–C). Of note is that deletion of just the Stx2-encoding prophage resulted in a significant increase in T3S and LEE1 expression levels but that the further deletion of the Stx1-encoding prophage had no further impact on expression, indicating that the repression is associated with the Stx2-encoding prophage only in EDL933. The impact of Stx phage deletion was further confirmed by RT-PCR (figure S1), with significantly higher *espD* transcript levels associated with either the single or double prophage deletions. Taken together, it is evident that the presence of Stx2 prophages in the EHEC O157 chromosome can limit T3S by repressing levels of LEE1 transcription. However, other uncharacterized changes between our laboratory stocks of EDL933 and TUV93-0 may also contribute to T3S differences between these strains.

**Table 1:** Bacterial strains associated with the study.

Strain	Details	Source
ZAP1238	*E. coli* O26 Stx2+	[47]
ZAP1239	*E. coli* O26 Stx2−	[47]
EDL933	*E. coli* O157:H7 EDL933	Lab stock
	Stx1+ Stx2+	
TUV93-0	EDL933 with excised Stx1 and Stx2 phages	[46]
ZAP1321	EDL933 *z1449(cII_stx2_)<>sacBkan*. Constructed by allelic exchange using pXLS13 (table S2)	This study
ZAP1322	EDL933ΔBP-933W (Stx2 phage) by allelic exchange of ZAP1321 using pXLS15 (table S2)	This study
ZAP1323	ZAP1321Δ*z1449*	This study
ZAP1324	ZAP1323 *z3357<>sacBkan*	This study
ZAP1325	ZAP1324Δ*z3357*	This study
ZAP1326	ZAP1322 CP-933V<>*sacBkan*	This study
Sakai *stx2A*::*kan*	Sequenced *E. coli* O157:H7 Sakai strain with a kanamycin cassette replacing the *stx2A* toxin gene (Sp5 *stx2A*::*kan*)	[67]
TUV(Sp5)	TUV93-0 containing a marked Stx2 phage conjugated from JC5029(Sp5) as described in Materials and Methods	This study
AAEC185	Laboratory K-12 strain for plasmid cloning	[70]
*E. coli* K12 EDCM367 (Nal^R^)	Spontaneous NalR derivative from *E. coli* K12 MG1655.	Provided by Prof. M. Masters, [68]
*E. coli* EDCM367(Sp5 *stx2A*::*kan*)	Generated by transduction from the Sakai *stx2A::kan* strain	This study
JC5029	Hfr strain	*E. coli* Genetic Stock Center
JC5029(Sp5 *stx2A*::*kan*)	Generated by transduction from the Sakai *stx2A::kan* strain	This study
TUV93-0Δ*gadE*	TUV93-0 with *gadE* replaced by allelic exchange	[14]
*E. coli* O157:H7 PT21/28 and PT32 bovine isolates	38 strains isolated as part of an international partnership in veterinary epidemiology (IPRAVE) project 2001–2006. Further information is provided in table S1.	[42]
*E. coli* O157:H7 PT21/28 and PT32 human isolates	24 strains provided by Dr. Lesley Allison from the *E. coli* reference laboratory, Edinburgh. Further information is provided in table S1.	Laboratory stocks

To examine if Stx2 prophage deletion has an effect on T3S in another EHEC serotype, two pairs of published *E. coli* O26:H11 strains were obtained (table 1). Both pairs are considered isogenic apart from the presence and absence of Stx2 prophage [47], the prophage were lost during culture of the bacteria. For one pair, T3S increased significantly in the absence of Stx2-encoding prophage (figure 2A), while for the other no T3S was detectable for either strain under the conditions tested (data not shown). For the pair with detectable T3S, the LEE1-GFP reporter was transformed into both strains and levels of expression measured throughout the growth curve (figure 2D). Again the presence of the Stx2 prophage correlated with significant repression of LEE1 expression in agreement with the Western blotting profile for EspD secretion and production of the T3S apparatus protein, EscJ (figure 2A–C).

### Repression of LEE1 promoter activity in *E. coli* K12 transduced with a Stx2 bacteriophage

**Figure 3:**
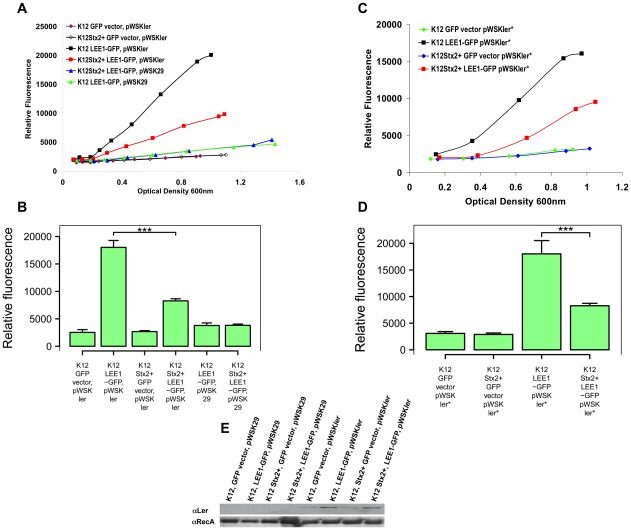
An Stx2-prophage represses LEE1 activation in *E. coli* K-12. Induction of the LEE1-promoter in isogenic *E. coli* K-12 strains with and without an integrated Stx2 (Sp5) phage. (A) A marked Stx2 phage was transduced into *E. coli* K-12 MG1655 as described in Materials and Methods. Expression of the LEE1-GFP reporter, or a control GFP plasmid (GFP vector) were compared in the Stx+ and Stx− K-12 strains with and without induction of the LEE-encoded regulator (pWSKler) or control plasmid (pWSK29) with 1 mM IPTG. (B) This experiment was repeated four times and fluorescence values determined at OD_600_ = 0.9 with the mean values and 95% confidence intervals presented. Stx2 lysogeny significantly reduced LEE1-GFP expression levels (*p*<0.001 (***)) following Ler induction (black and red squares), but there was no significant difference without Ler induction (green and blue triangles). (C–D) The experiments as above were repeated with a 6×His-tagged Ler construct (pWSKler*) to enable a comparison of Ler levels in the different backgrounds. Again the Stx2 lysogen led to significantly reduced levels (*p*<0.001 (***)) of population fluorescence following Ler induction (red and black squares in (C) when compared at OD_600_ = 0.9 in (D - presented as mean and 95% confidence intervals). (E) 6×His-tagged Ler levels were compared with RecA in the different strains by Western blotting from whole cell samples taken at OD_600_ = 0.9. Both strains containing the LEE1-GFP fusion and the induced pWSKler* contained equivalent levels of the induced 6×His-tagged Ler protein, lanes 6 & 8.

In order to determine whether the repression of T3S expression by Stx2 prophages could also be detected in a distinct genetic background, the marked Stx2 bacteriophage (Sp5) induced from *E. coli* O157:H7 Sakai was used to transduce *E. coli* K12 MG1655. This transduction was achieved and the establishment of a lysogen in *E. coli* K-12 confirmed by selectable markers and PCR assays (Materials and Methods). This *E. coli* K-12 Stx2 prophage lysogen and the parental MG1655 strain were then transformed with the LEE1::GFP promoter fusion plasmid and fluorescence levels measured throughout the growth curve (figure 3A). While there was some detectable expression from the LEE1 promoter there was no significant difference in LEE1 expression levels between the two strains (figure 3A). However, neither of these strains contains the LEE encoded regulator (Ler) which is known to activate T3S by relieving H-NS repression of LEE1 and other LEE promoters. To examine the effect of the Stx2 prophage on Ler-induction of LEE1 expression, *ler* was expressed from an IPTG-inducible promoter on pWSK29 in the same pair of *E. coli* K-12 strains containing the LEE1 reporter. Induction of Ler was carried out using IPTG and fluorescence levels measured throughout the growth curve. As anticipated expressing Ler *in trans* significantly increased expression from the LEE1 promoter fusion in the *E. coli* K-12 strain (figure 3A). However, this *ler*-dependent activation did not occur to the same extent in the *E. coli* K-12 derivative containing the Stx2 prophage (figure 3A). This result implied that Stx2 prophage-based repression of T3S involved inhibition of Ler-mediated LEE1 promoter activation. To confirm that Ler was induced by IPTG induction to a similar level in the two backgrounds, a modified Ler was expressed from pWSK29 with a carboxy-terminal six Histidine tag (6×His-Ler) to allow levels to be determined by Western blotting. This construct was also able to induce LEE1 expression in *E. coli* MG1655 and again this activation of transcription from the LEE1 promoter was repressed by the presence of the marked Stx2 prophage (Sp5) from Sakai (figure 3B). Whole cell protein samples were blotted for detection of 6×His-Ler and the isogenic K-12 strains containing the LEE1-GFP reporter (figure 3E, lanes 6 & 8) contained equivalent levels of the tagged Ler protein when compared to RecA levels as a control. This implied that although Ler was induced and present its capacity to act on the LEE1 promoter was limited by the presence of the integrated Stx2 phage in the chromosome.

### The lysogeny regulator CII can repress type III secretion

**Figure 4:**
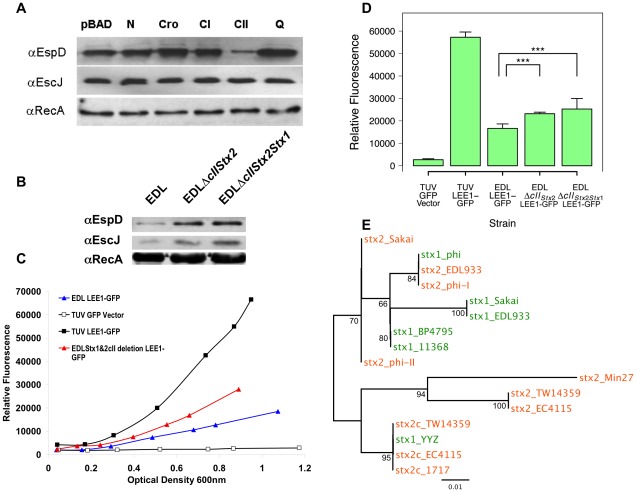
The CII protein is involved in repression of T3S in EHEC O157. (A) The defined bacteriophage lysis/lysogeny regulators were cloned into the pBAD18 vector and induced in *E. coli* TUV93-0 that does not contain any Stx prophages. Western blot detection of EspD from bacterial supernatants and EscJ & RecA from whole cell samples. (B) Western blot analysis of the same proteins from *E. coli* EDL933 and isogenic strains from which the Stx2 *cII* gene and then the Stx1 phage *cII* gene had been deleted by allelic exchange as described in Materials and Methods. (C–D) Population fluorescence from the LEE1-GFP reporter, or control plasmid, were examined in EDL933 and *cII* deletion strains and presented in D as mean and 95% confidence intervals. Deletion of either the single *cII* from the Stx2-encoding bacteriophage or both *cII* genes led to significant increases (p<0.001 (***)) in population fluorescence levels at OD_600_ = 0.9. (E) CII phylogenetic analysis. Phylogram shows unrooted maximum-likelihood tree of 16 publicly available *cII* nucleotide sequences from Stx1 (green) and Stx2/Stx2c (orange) encoding bacteriophages. Abbreviations: EDL933, EC4115, Sakai, and TW14359 (from *E. coli* O157:H7 complete genomes); 11368 (from *E. coli* O26:H11 complete genome); BP-4795 (phage BP-4795 from *E. coli* O84:H4 str. 4795/97); Min27 (phage Min27 from *E. coli* O157:H7 str. Min27); phi and phi-II (phages Stx1phi and Stx2phiII from *E. coli* O157:H7 str. Morioka V526, respectively); phi-I (phage Stx2phiI from *E. coli* O157:H7 str. Okoyama); YYZ (phage YYZ-2008 from *E. coli* O157:H7 str. EC970520); and 1717 (Stx2-converting phage 1717 from unspecified strain of *E. coli* O157:H7). Scale bar shows substitutions per site.

One explanation for the failure of Ler to induce LEE1 expression in the presence of the Stx2 lysogen is that a regulator or regulators expressed from the Stx2 lysogen repress Ler activity on the LEE1 promoter. Several of the key lysis/lysogeny regulators characterized for phage lambda were cloned from the Sakai Stx2 phage (Sp5) into the arabinose-inducible vector pBAD myc HisA (table S2). These clones were sequenced and confirmed as identical to the published genes from Sp5. The clones were electroporated into the Stx phage negative *E. coli* O157:H7 strain TUV93-0 and the T3S profiles determined from supernatants following arabinose induction (figure 4a). Only the clone of *cII* showed any evidence of T3S repression. However, it is established that CII induction can be toxic for cells and we observed reduced growth rates when inducing expression of *cII*. It is also apparent that in this case, even though EspD levels were reduced, EscJ expression was not, indicating a difference over the Stx2 prophage repression phenotype. Of note was the same reduced growth rate when *cII* was induced in EPEC O127 E2348/69, but in this case there was no repression of T3S (data not shown), indicating a regulatory difference in this Stx phage negative background. To determine the relevance of *cII* to repression of T3S at physiological levels, the *cII* gene was deleted sequentially from the Stx2 prophage and then from the Stx1 prophage in *E. coli* O157 EDL933. Western blotting for T3S proteins as well as fluorescence levels from the LEE1-GFP reporter transformed into the strain demonstrated significantly increased LEE1 expression and T3S in the absence of *cII* from the Stx2 prophage (figure 4A–B), but with no further increase in secretion following the deletion of the Stx1-prophage *cII* regulator. This result is consistent with the results obtained following sequential deletion of the Stx2 and Stx1 prophages from EDL933 (figure 2). Available database *cII* sequences from Stx1- and Stx2-encoding bacteriophages were aligned and clustered (figure 4E). This indicated that while *cII* sequences can group to some extent with Stx type, identical *cII* sequences can also be associated with both Stx1 and Stx2-encoding phages. In the case of EDL933, the BP933W (Stx2)-associated CII peptide (Z1449) shares 94.6% amino acid identity to the predicted CII peptide (Z3357) sequence from CP-933V (Stx1) which could account for the differences in activity observed in this study. Both EDL933 and Sakai also encode an additional gene within syntenic, non-*stx* associated lambdoid phages (*z0310* in EDL933 CP-933H prophage and *ecs0276* in Sakai Sp1 prophage, respectively), with low predicted identity to the Stx-associated CII homologs (<30% amino acid identity with the EDL933 Stx2 prophage CII peptide sequence). Table S4 summarizes the results of these comparisons.

### Exogenous expression of homologous *cI* represses Stx prophage regulation of T3S

**Figure 5:**
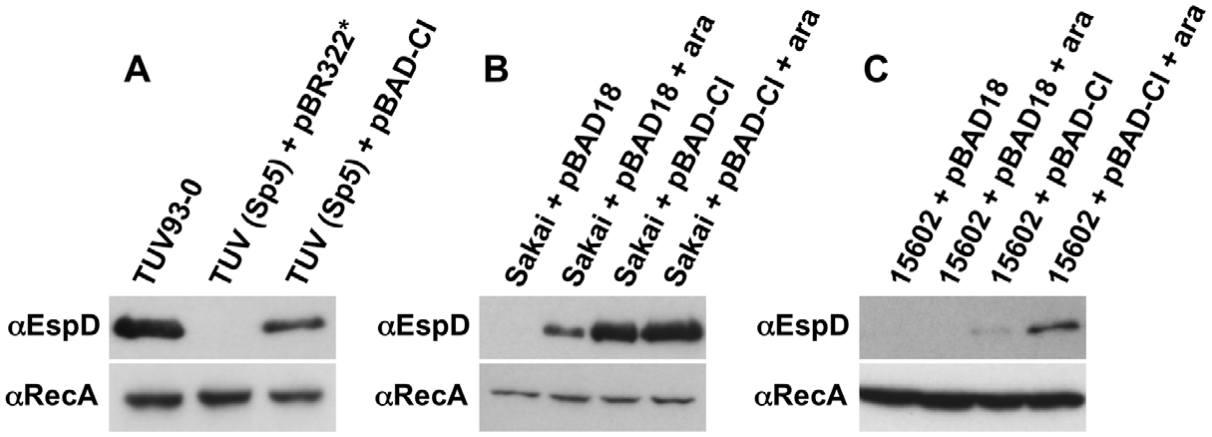
Homologous *cI* expression represses prophage control of T3S. Western blot of Secreted EspD and whole cell pellet RecA levels (preparation control) for the indicated strains: (A) Conjugation of a marked Sp5 Stx2-prophage from *E. coli* O157 Sakai was carried out into TUV93-0 containing an induced clone of Sp5 *cI* to prevent zygotic induction. This pBAD-CI clone was then displaced with a variant of pBR322. The *cI* clone limited the repressive impact of the Sp5 prophage on T3S. (B) *E. coli* O157 Sakai was transformed with both pBAD18 and pBAD-CI. The presence of pBAD-CI with or without arabinose induction increased the level of EspD secretion. (C) An *E. coli* O157 PT21/28 isolate (15602, table S1) was transformed with both pBAD18 and pBAD-CI. The presence of the pBAD-CI clone led to detectable EspD secretion that was increased on induction of *cI*. There was no increase in T3S when the same plasmid was induced in *E. coli* O157 EDL933 (data not shown), indicating that the induction may be specific to strains containing a cognate *cI* on the Stx2-encoding bacteriophage.

In the case of classic lambda, CII and the majority of prophage regulators are silenced by CI once lysogeny has been established [4], [5]. Given that CII and potentially other regulators on Stx2/2c prophages are involved in the repression of T3S when the prophage is in a lysogenic state, we wanted to determine whether increased expression of a homologous *cI* could alleviate this prophage-based control by repressing the expression of these controlling factors such as *cII*. This was tested in a number of ways:

Firstly, when the marked Sakai Sp5 Stx2-prophage was conjugated into TUV93-0 (figure 2) this required that *cI* from this phage was cloned, transformed and expressed in TUV93-0 in order to prevent zygotic induction of phage-mediated lysis on receipt of the transferred prophage. It had already been demonstrated that this clone had no impact on T3S in TUV93-0 in the absence of a Stx2 prophage (figure 4), but when this *cI*-encoding plasmid was subsequently displaced from the TUV93-0(Sp5) background, there was further suppression of T3S as determined by a reduction in EspD secretion levels (figure 5A). The presence of the CI-encoding plasmid was therefore limiting the capacity of the bacteriophage to repress T3S.

Secondly, the same Sakai (Sp5) *cI* construct was then transformed into both *E. coli* O157 Sakai and EDL933 backgrounds. While there was no detectable impact of the regulator on T3S in EDL933 (data not shown), the expressed regulator increased levels of T3S in the Sakai background (figure 5B). This was consistent with the fact that the Sakai Sp5 produces a CI regulator that is markedly different (52/217 identity, 24%) to that encoded by the Stx2 prophage (BP-933W) of EDL933.

Finally, in order to test whether the prophage-based regulation contributed to repression of T3S in the original PT21/28 isolates that contain both Stx2 and Stx2c prophages (figure 1), the Sakai Sp5 *cI* clone was transformed into the PT21/28 strain 15602 (table S1). Sequencing of amplified *cI* alleles from this strain (Materials and Methods) indicated that it contains an identical *cI* to that present in Sakai Sp5. The presence of the Sp5 *cI* clone and further induction with arabinose increased EspD secretion in this PT21/28 strain (figure 5C), supporting the concept that increased expression of a homologous *cI* can counteract the activity of Stx2 prophage-based regulators, including *cII*, that repress T3S.

## Discussion

Enterohemorrhagic *Escherichia coli* strains are characterised by the integration of lambdoid-like phages into their chromosome that encode Shiga toxins [1], [8], [47], [48]. EHEC O157 strains can be lysogenized by different types of Stx phage, but those associated with Stx2 and Stx2c are considered the most important in terms of human virulence [49], [50]. Some strains carry multiple Stx phages indicating differences in phage exclusion control beyond that characterized for classical lambda [51], [52]. A key question is whether Stx phage lysogeny confers an evolutionary advantage on the recipient EHEC strain, and whether this might underpin the epidemiology of EHEC O157 strains? A large epidemiological study of farms in Scotland demonstrated that PT21/28 strains were more likely to be associated with higher level fecal counts compared with PT32 strains. In addition, PT21/28 strains over that period were the most prevalent phage type associated with human infection [41], [42]. The initial aim of this study was to compare PT21/28 and PT32 strains in terms of genomic differences to determine how these inform the epidemiology.

Comparative genomic hybridisation (CGH) and PCR analyses demonstrated that Stx phage carriage differed between the phage types. PT21/28 strains generally carried both Stx2 and Stx2c bacteriophages, irrespective of whether the strains were sourced from cattle or humans. By contrast, nearly all the bovine PT32 strains analysed carried only the Stx2c phage. The situation for the limited number of human PT32 strains was more complex, but these were significantly more likely to contain a Stx2 phage in comparison to the bovine PT32 strains. This data indicates that strains carrying both Stx2 and Stx2c phages are more likely to be associated with human infection. This result is in line with recently published research on Clade 8 strains in the USA that are considered to be more virulent. Clade 8 strains were significantly more likely to carry both the *stx2* and *stx2c* genes in comparison to the other clades containing *stx2c* [40]. All clade 8 strains tested had *stx2*, and 57.6% had *stx2c*. Whether this association for both relates to differences in toxicity of the strains or other phenotypes associated with the strains is not known, although more recent work has indicated that levels of Stx2 production alone are likely to be important, irrespective of lineage, in terms of association with human clinical disease [49].

As type III secretion (T3S) is essential for cattle colonisation and variation in regulation may be associated with differences in shedding level from cattle [44], T3S levels of PT32 and PT21/28 strains was investigated. Levels of T3S varied markedly between strains in agreement with our previous work on T3S profiles of EHEC O157 strains [53]. T3S levels were low in the majority of PT21/28 strains compared with the PT32 strains. Extensive research has defined multiple regulatory inputs for T3S and it is likely that the variation observed will be due to a complex combination of alleles. For example, use of a LEE1 reporter readout in different lineages of EHEC O157 strains has yielded a complex and widely distributed pattern of expression with no clear association with particular integrated O-islands or S-loops [54]. However, the finding in the present study that strains with both Stx2 and Stx phages had on average lower levels of LEE1 expression than strains with a single type of Stx2 bacteriophage prompted us to test strain pairs with and without Stx bacteriophages. Analysis of these strains demonstrated that Stx2 phage insertion into the chromosome leads to repression of T3S, this was also apparent in a pair of O26 strains, including one from which the Stx2 phage had been naturally cured [47]. As stated there are likely to be multiple variable inputs into T3S expression and this was confirmed by analysis of the potentially isogenic EDL933 and TUV93-0 strains. While Stx2 bacteriophage insertion and deletion repressed and activated T3S respectively, other differences between the strains may also account for the marked difference in T3S levels between these strains.

To confirm and study the Stx2 bacteriophage repression in another background, the sequenced *E. coli* K12 MG1655 strain was lysogenised with a marked Stx2 phage from the sequenced *E. coli* O157:H7 Sakai strain. This set of experiments provided insight into the mechanism of the Stx2 prophage control as the capacity of Ler to induce LEE1 expression was reduced in the presence of the Stx2 lysogen despite equivalent levels of the Ler protein being present. In turn, this will limit expression of the other LEE operons under Ler control reducing T3S levels from the EHEC lysogen. As LEE1 expression is subject to auto-regulation by Ler, we cannot rule out repression of LEE1 transcription independently of the inhibition of Ler activation.

Of note is recently published work examining the impact of Stx phage insertion on gene expression in *E. coli* K-12 by micro-array [55]. There were 166 genes found to be differentially expressed indicating global regulation by the integrating prophage. 62 transcripts were down-regulated and 104 up-regulated, including motility and acid-resistance associated genes. This included increased expression of *gadE* that is known to be involved in repression of T3S so increased *gadE* expression following Stx phage lysogeny could account for some of the reduced expression [14], [56], [57]. In the present study, this was investigated in *E. coli* K-12, in which Stx2 lysogeny was demonstrated to increase *gadE* expression (figure S2A), but in an *E. coli* O157 background, the deletion of *gadE* had no impact on the capacity of the integrated Stx2 prophage to repress T3S (figure S2B), indicating that while this pathway may be relevant for the prophage control, it is not required.

Previous work has established that the LEE2/3 operons, but not LEE1 are subject to repression by LexA in EPEC with activation following SOS induction [58]. The repression of Ler activity on LEE1 demonstrated in the present study may be the result of established lambda lysogeny regulators and/or regulators encoded elsewhere in the bacteriophages. To test established lambda phage regulators, *cI*, *cII*, *cro*, anti-terminators N and Q were cloned and induced in TUV93-0. CI was a potential regulator as this is the main phage encoded-protein known to be expressed constitutively during lysogeny [52] but its induction had no direct impact on T3S in the absence of an integrated Stx prophage. CII was the only induced regulator that had a direct effect in this background, and de-regulated CII expression is known to have effects on growth rate [59] and so the T3S changes may have been due to indirect effects. For example, ClpX activity is increased in stressed bacteria and this is known act predominately on LEE4/5 and so would affect T3S levels of the LEE4-encoded EspD but not EscJ expressed from LEE3 [60]. To test the potential importance of *cII*, both copies were sequentially deleted from the Stx2- and then Stx1-encoding prophages in EDL933. T3S was increased following deletion of *cII_Stx2_* but no further increase was measured on further deletion of *cII_Stx1_*, indicating a role of *cII_Stx2_* in the control of T3S regulation. This result is however incongruous with the established regulation and function of CII, which is thought to primarily aid the establishment of the lambda lysogen [52]. While this is the case for bacteriophage lambda there may well be differences in the regulation between classic lambda and Stx lambdoid bacteriophages; for example it is already clear that these phages are tolerant of multiple lysogens [51]. Further confirmation that the repression of T3S is controlled by Stx2 phage-based regulators such as CII that are under the control of CI was shown by increasing expression of a cognate *cI* in a number of EHEC backgrounds which led to an increase in T3S levels (Figure 5A–C). We cannot rule out that other Stx2 bacteriophage-based regulators may also contribute to repression of T3S following lysogeny but both *cI* and *cII* sequence variation, as well as differences in their expression and potential to cross-talk with other Stx-encoding prophages are likely to account for the different repressive capacities of Stx-encoding phages and T3S variation between EHEC strains.

**Figure 6:**
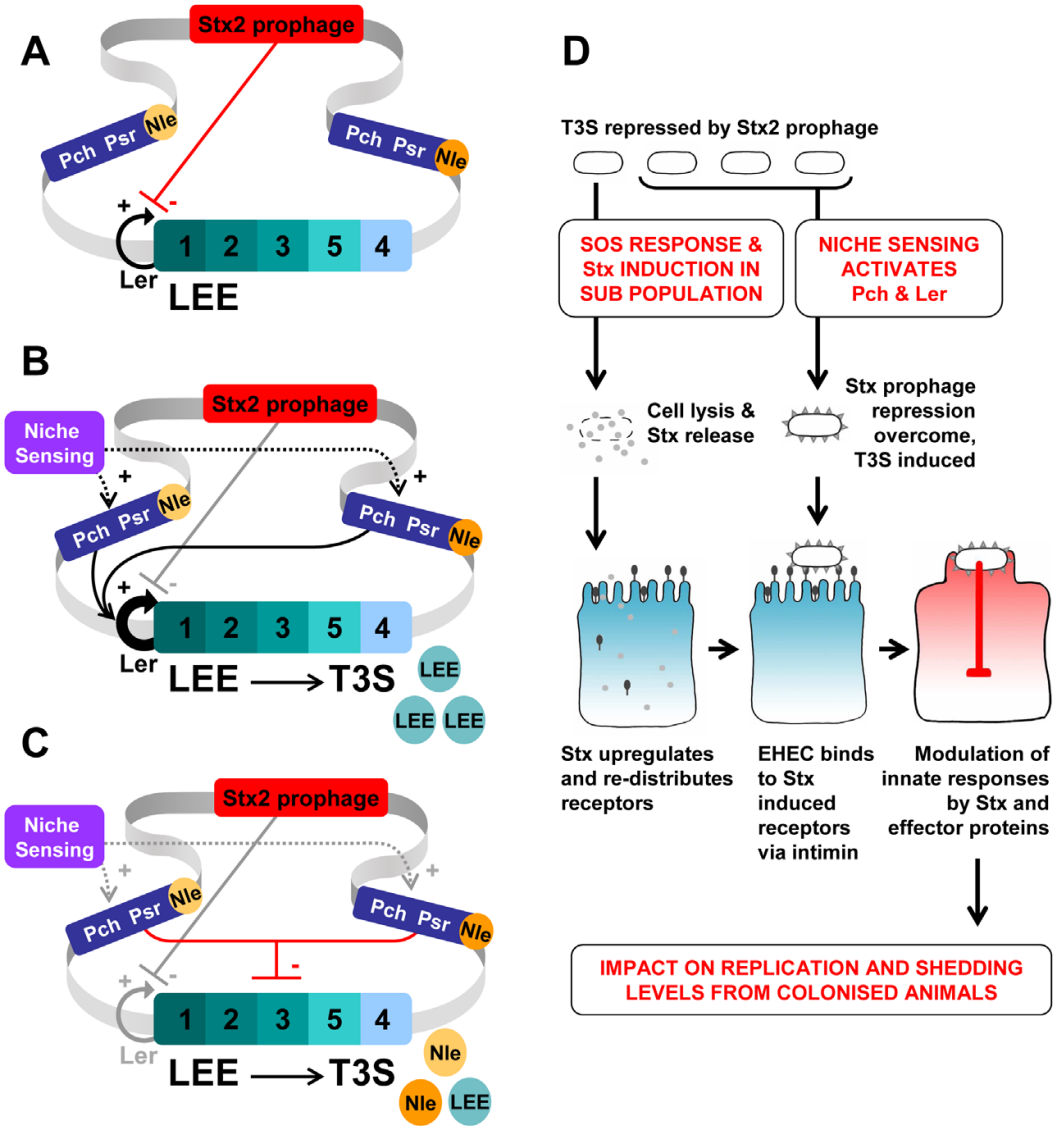
Regulatory scheme for prophage control of T3S and impact on cattle colonisation. (A) Schematic diagram showing that the integrated Shiga toxin prophage represses type III secretion (T3S) by restricting Ler-mediated LEE promoter activation. Under the conditions shown there is no expression of the PchA/B regulators associated with other integrated prophages that express effector proteins secreted by the T3S system. (B) Repression is overridden by the activity of activators such as PchA/B that are induced following sensing of niche specific signals in the animal host. A number of environmental signals are known to control T3S [13] including quorum sensing [63] which is proposed to contribute to the tropism of EHEC O157 for the terminal rectum of cattle [43]. However, much less is known about whether these can act through Pch activation. PchA/B stimulate LEE1 and Ler expression leading to production of the T3S apparatus and secretion of LEE-encoded regulators, indicated as blue circles marked ‘LEE’ in the figure. (C) Psr regulators on effector-encoding prophages increase *gadE* expression leading to repression of LEE-encoded effector protein secretion. It is proposed that this prophage regulation allows non-LEE encoded effectors (Nle) to compete for export through the T3S system [14]. (D) A model for EHEC interaction with the epithelium. SOS stress responses result in prophage induction and Stx release in a subset of the population. Potentially certain stresses associated with the interaction with epithelial cells may induce this response. The released toxin induces the expression and redistribution of receptors to the epithelial cell surface [32], [62]. T3S is repressed but can be induced by Pch regulators [12], [13], RgdR [15] and further controlled by PsrA/B [14] present on cryptic prophages to ensure co-ordinate T3S apparatus expression and effector protein secretion (figure 5A–C). The induction of T3S includes intimin expression on the outer membrane of the bacteria allowing binding to Stx-induced receptors, including nucleolin [32]. This leads to intimate attachment and lesion formation. Secreted effector proteins can repress inflammation as can Stx [33]–[39]. It is proposed that the degree and nature of this modulation will be different between strains impacting on bacterial replication and therefore the extent of excretion from the animal.

So why is this repression of T3S associated with Stx phage lysogeny? We propose that Stx phages repress LEE1 expression to effectively take control of this critical colonisation factor. The Stx bacteriophage, through this regulation, may ensure that bacterial colonisation and persistence become dependent on the bacteriophage. It is interesting that EHEC strains, by comparison with the sequenced EPEC strain O127:H6 have a greater number of T3S effector proteins expressed from a variety of cryptic prophages [8], [61]. These effector proteins have their expression co-ordinated with production of the T3S system by the PchA/B regulators and Ler that act on both effector protein expression and LEE1 [12], [13]. We propose that the repression demonstrated by Stx2 phages could select for co-integration of effector-encoding prophages and their associated LEE regulators (figure 6A–C). As a consequence, it is the network of prophage-based repressors and activators controlling T3S that is important for EHEC strains for which Stx2 prophages must also be taken into consideration [13].

It is also evident that Stx phages provide a selective advantage in EHEC strains for colonisation and persistence through the production of Shiga toxins. SOS induction in the gastrointestinal tract will lead to lysis of a subset of the bacteria with Stx release (figure 6D). Stx allows increased expression and relocation of nucleolin and other receptors to the epithelial cell surface where they bind to the EHEC adhesin, intimin promoting EHEC colonization [32], [62]. This function of Stx directly links Stx phage regulation and LEE-promoted adherence, as intimin is expressed from the LEE and is necessary for intimate attachment and attaching and effacing (A/E) lesion formation by binding to the translocated intimin receptor (Tir) also expressed from the LEE and secreted by the T3S system. It is established that EHEC strains vary markedly in their capacity to produce Stx toxins [49], a phenotype that is intricately associated with Stx phage carriage and levels of lysis induction. EHEC strains also vary in their capacity to secrete T3 translocon and effector proteins [45], [53] a phenotype that can now also be related, in part, to the combination of Stx phages carried by a strain. The heterogeneous nature of lysis induction means there is a subset of bacteria that do not lyse and which can then induce T3S when appropriate niche signals are detected, including quorum sensing [63]; these bacteria benefit from Stx priming of the epithelium as a result of the lysed population (figure 6D). In this way, the Stx phage and effector prophage repertoire of an EHEC strain are critical factors governing colonization of the gastrointestinal tract and subsequent innate responses that will determine excretion levels from the animal (figure 6D). Future work will examine whether increased excretion of strains from cattle is associated with integration of multiple Stx phages, altered T3S regulation and increased Stx toxin levels, with important ramifications for the selection of strains able to cause more severe infections in humans.

## Materials and Methods

### Bacterial strains, plasmids, oligonucleotides and media

The bacterial strains and plasmids used in the study are described in tables 1, S1 and S2. Table S3 lists the oligonucleotide primers used. MEM-HEPES is minimal essential medium with HEPES buffer (Sigma), containing additional glucose to a final concentration of 0.2%. LB broth was also used (Oxoid). Antibiotics were included when required at the following concentrations: chloramphenicol (C) 12.5 µg/ml, Nalidixic acid (20 µg/ml) kanamycin (K) 25 µg/ml and ampicillin (A) 50 µg/ml, spectinomycin (50 µg/ml) and tetracycline (15 µg/ml).

### Cloning of prophage-encoded regulators

*ler*, *cII* alleles, *cI*, *cro*, anti-terminator N and Q genes were cloned from *E. coli* O157 Sakai and EDL933 strains as explained in table S2 with primer sequences described in table S3. These clones were confirmed by sequencing (GATC Biotech). The *cI* alleles from 15602 were sequenced following amplification using CIseq1 and CIseq2 primers (table S3). Clones in pBAD/HisA were induced with 0.2% (w/v) L-arabinose when indicated.

### Comparative genomic hybridization (CGH)

Genomic DNA was extracted with Invitrogen ChargeSwitch gDNA Mini Bacteria Kit (Invitrogen). Labeling was carried out with Bioprime Plus Array CGH Genomic Labelling System (Invitrogen). Protocols for pre-hybridization, hybridization and washing for ultragaps slides were from UBEC project (School of Biosciences, University of Birmingham). The arrays were 70-mer spotted oligonucleotides based on open reading frames from *E. coli* K-12, *E. coli* Sakai, and *E. coli* EDL933. Briefly, array slides were washed twice in Wash Buffer II (0.1% SDS, 0.1× SSC), each time for 30S. Then slides were transferred in pre-hybridization buffer (0.1% BSA, 0.1% SDS, 5× SSC) for 120 min. After washing in Wash Buffer II and III (0.1× SSC), the denatured hybridization probe mixture (30% formamide, 5× SSC, 0.1% SDS, 0.1 mg/ml Salmon sperm DNA, 1× Denhardt's Solution and 80 picomoles Cy3 and Cy5 probe) was added to slides and incubated overnight. Next day, the array slides were washed in Wash Buffer I (0.1% SDS, 2× SSC), II and III and scanned with an Axon scanner. The hybridization levels were analyzed using Genespring GX 7.3.1 (Agilent).

### PCR to identify *stx2-* and *stx2c-encoding* bacteriophages

Genomic DNA for 62 tested strains was extracted with the Invitrogen ChargeSwitch gDNA Mini Bacteria Kit. The concentration of the genomic DNA was measured by a nanodrop. The method of Wang *et al.* [64] was used to determine the presence of *stx2* and *stx2c* coding sequences. 100 ng of genomic DNA was used for each PCR reaction. An amplicon of 115 bp with the respective primers indicated the presence of an Stx2-encoding bacteriophage while an amplicon of 124 bp with the *stx2c*-specific primers indicated the presence of an Stx2c-encoding prophage.

### Preparation of secreted proteins and bacterial fractions for protein analyses

Bacteria were cultured in 30 ml of MEM-HEPES at 37°C (200 r.p.m.) to an OD_600_ of 0.8 unless specifically stated. The bacterial cells were pelleted by centrifugation at 4000× *g* for 15 min, and supernatants were passed through filters (0.45 µm). Supernatant proteins were precipitated overnight with 10% TCA, and separated by centrifugation at 4000× *g* for 30 min (4°C); the proteins were suspended in 100 µl of 1.5 M Tris (pH 8.8). The bacterial pellet was initially suspended in 50 µl Laemmli sample buffer (Sigma) and 50 µl water. Proteins were separated by SDS-PAGE using standard methods and Western blotting performed as described previously [45], [65]. Primary antibodies for Western blotting were raised against the proteins described with the source provided in brackets: α-EspD (Gift from Prof. T. Chakraborty, University of Giessen), α-EscJ (Gift from Dr Ando and Prof. Tobe, Osaka University), α-RecA (Stressgen/Enzo Life Sciences).

### Quantification of population fluorescence levels

Strains were electro-transformed with pAJR70–71 (table S2) and the resultant transformants were cultured overnight in LB broth and then diluted 1∶100 into fresh, MEM-HEPES with appropriate antibiotics. Typically, 30 ml was cultured in Erlenmeyer flasks shaken at 200 rpm, 37°C. The optical density of the cultures was monitored by determination of the OD_600_. Cultures were sampled every hour for optical density and GFP measurement. The total fluorescence produced by the population was determined by analyzing 100 µl aliquots of culture with a fluorescent plate reader (Fluostar Optima; BMG). Promoterless plasmid pAJR70 acted as a control for background fluorescence.

### RT-qPCR

Bacterial RNA was purified with an RNeasy kit (Qiagen) and reverse transcribed with random primers Affinityscript (Stratagene). qPCR was carried out with a PowerSybr mastermix (Applied Biosystems). The qPCR primers used are listed in table S3. Transcript abundance was normalized to 16S rRNA and relative transcription calculated using MxPro software (Stratagene).

### Constructs for allelic exchange

In order to construct plasmids for chromosomal exchange, flanking regions of the *z3357*, *z1449* (*cII_stx2_*) genes and Stx1 and Stx2 phages were PCR amplified and cloned into the temperature-sensitive plasmid pIB307 (table S2). Primer pairs as described in table S3: z3357 up 5′, z3357 up 3′ and z3357 down 5′, z3357 down 3′; *z1449* up 5′, *z1449* up3′ and *z1449* down 5′, *z1449* down 3′; stx1 up 5′, stx1 up3′ and stx1 down 5′, stx1 down 3′; stx2 up 5′, stx2 up3′ and stx2 down 5′, stx2 down 3 were used to amplify *z3357*, *z1449* Stx1 phage and Stx2 phage flanking regions sequences from *E. coli* O157:H7 EDL933. These products were cleaned with a Invitrogen PCR purification kit, digested with *BamH*I and *Hind*III or *Sac*I, or *Sal*I, re-cleaned, and then ligated with digested pIB307 (table S2) to obtain pXLS10, pXLS11, pXLS12 and pXLS15 containing flanking regions of *z3357*, *z1449*, *stx1* phage, and *stx2* phage respectively. To produce plasmids for exchange, a *sacBkan* cassette was cloned into the *BamH*I sites of pXLS11, pXLS10 and pXLS12 creating pXLS13, pXLS14 and pXLS16 that would allow the chromosomal replacement of *z1449*, *z3357* and *stx1* genes respectively with a selectable marker and a counter-selection gene.

### Allelic exchange

The method of Emmerson *et al.* was used for allelic exchange [66]. Plasmid pXLS13 (table S2) was transformed into EDL933 to obtain the intermediate strain with *z1449* (*cII_stx2_*) replaced by *sacBkan* cassette. Briefly, pXLS13 was electroporated into EDL933 (table 1) and cultured at 30°C on LB-C plates. Ten transformants were inoculated into pre-warmed LB-C at 42°C and passaged repeatedly in LB-C broth at 42°C to obtain co-integrates. The culture was further passaged at 30°C in LB-K broth to select for the complete exchange. The kanamycin resistant and chloramphenicol sensitive strain was confirmed by PCR with primer pairs z1449 5′ and z1449 3′ for inside of *z1449*, z1449 external 5′ and z1449 external 3′ for outside of *z1449*, sacB 5′ and sacB 3′ for *sacBkan* cassette (table S3). The resultant strain was termed ZAP1321. ZAP1321 was then transformed with plasmids pXLS15 and pXLS11 and allelic exchange carried out to generate strains with Stx2 phage and *z1449* (*cII_stx2_*) clean deletions respectively. These allelic exchanges were as above except that the cultures at 30°C did not contain any antibiotic selection. The required clones were identified by sensitivity to both kanamycin and chloramphenicol and confirmed by PCR using primer pairs: wrbA and intW plus z1503 and z1504 for the junction sites of Stx2; stx2 5′ and stx2 3′ for a region inside the Stx2 phage; z1449 5′ and z1449 3′ for inside of *z1449 (cII_stx2_)*, z1449 external 5′ and z1449 external 3′ for outside of *z1449*, sacB 5′ and sacB 3′ for *sacBkan* cassette. The clean Stx2 prophage and z*1449* deletion strains were termed ZAP1322 and ZAP1323 respectively (table 1). In order to construct a strain deleted for both Stx1 and Stx2 phages, ZAP1322 was used to generate a strain with the Stx1 prophage replaced with the *sacBkan* cassette using pXLS16 and the exchange protocol as above. The strain was confirmed by resistance profile and PCR with appropriate primer pairs (table S3). The resultant strain was termed as ZAP1326 (EDLStx1&2^−^). To construct a strain with both *z1449 (cII_stx2_)* and *z3357* (*cII_stx1_*) deleted, ZAP1323 was used for allelic exchange. pXLS14 was transformed into ZAP1323 and the exchange carried out as described above to produce strain with *z3357* replaced by a *sacBkan* cassette. The strain was confirmed by antibiotic resistance profile and PCR with appropriate primer pairs (table S3). This strain was termed ZAP1324. ZAP1324 was electroporated with pXLS10 and the clean deletion of *z3357* (*cII_stx1_*) was obtained by allelic exchange and confirmed by resistance profile and PCR. The final *z3357* and *z1449* clean deletion was termed ZAP1325 (table 1).

### Strain construction by transduction and conjugation

A marked Sp5 bacteriophage from an Stx negative variant of *E. coli* O157:H7 Sakai containing a *stx2A*::*km^r^* cassette [67] was transferred to other strains by either transduction or conjugation. For transduction, the bacteriophage were prepared by culture of the strain in LB at 37°C followed by addition of 1 µg/ml mitomycin C (MMC) until lysed. The culture was centrifuged to pellet debris then chloroform used to extract bacteriophage from the supernatant and to kill any remaining cells. This bacteriophage preparation was used to transduce a spontaneous Nalidixic acid resistant strain of *E. coli* K12 EDCM367 [68]. Positive transductants were selected on LB agar plates containing kanamycin and then screened on nalidixic acid. The presence of the marked Stx prophage was further confirmed by PCR and MMC-induced lysis.

For conjugation into TUV93-0 or TUV93-0Δ*gadE* (table 1), Hfr JC5029 (*E. coli* Genetic Stock Center) strain transduced with the marked Sp5 as above was used. Spontaneous Nal^r^ TUV93-0 strains were selected. These and JC5029(Sp5) were transformed with pBAD-CI (table S2) to prevent zygotic induction following conjugation and to allow the donor to grow in the presence of ampicillin during the mating. Plate mating was carried out on LB agar+0.2% arabinose and ampicillin. The bacteria were washed off with LB and then selected on agar plates containing kanamycin and nalidixic acid. The absence of the Hfr strain was confirmed by sensitivity to spectinomycin to which JC5029 is resistant. The final strain was confirmed to contain the marked Sp5 by PCR and lysis following addition of mitomycin C (1 µg/ml). To remove pBAD-CI (table S2) from TUV93-0 (Sp5) or TUV93-0Δ*gadE* (Sp5), the strain was transformed with pBR322Δ*Sca*I-*Ssp*I with selection for tetracycline resistance and screening for sensitivity to ampicillin on LB agar plates.

### Phylogenetic analysis of CII

Phylogenetic analysis of 16 *cII* nucleotide sequences from publicly available Stx1- and Stx2/Stx2c-encoding bacteriophages was carried out by maximum likelihood as implemented in PHYLIP (http://www.phylip.com/). Bootstrap values were calculated from 100 replicates. Sequences were obtained from both complete bacterial genomes and complete phage genomes from the NCBI RefSeq database (source genome accessions and *cII* locus tag unique identifiers are listed with square and round brackets, respectively); i) *cII* sequences from complete bacterial genomes: *E. coli* O157:H7 str. EDL933 [NC_002655] Stx1 phage (Z1449) and Stx2 phage (Z3357), *E. coli* O157:H7 str. EC4115 [NC_011353] Stx2 phage (ECH74115_3554) and Stx2c phage (ECH74115_2923), *E. coli* O157:H7 str. Sakai (NC_002695) Stx1 phage (ECs2988) and Stx2 phage (ECs1187), *E. coli* O157:H7 TW14359 [NC_013008] Stx2 phage (ECSP_3270) and Stx2c phage (ECSP_2739), *E. coli* O26:H11 str. 11368 [NC_013361] Stx1 phage (ECO26_1586); ii) *cII* sequences from complete phage genomes: Stx1 phage BP-4795 [NC_004813] from *E. coli* O84:H4 str. 4795/97 (phi4795p23); Stx2 phage Min27 [NC_010237] from *E. coli* O157:H7 str. Min27 (pMIN27_28); Stx1 phage Stx1phi [NC_004913] from *E. coli* O157:H7 str. Morioka V526 (Stx1_gp56), Stx2 phage Stx2phiII [NC_004914] from *E. coli* O157:H7 str. Morioka V526 (Stx2II_gp57), Stx2 phage Stx2phiI [NC_003525] from *E. coli* O157:H7 str. Okoyama (Stx2Ip123); Stx1 phage YYZ-2008 [NC_011356] from *E. coli* O157:H7 str. EC970520 (YYZ_gp27); and Stx2 phage 1717 [NC_011357] from an unspecified strain of *E. coli* O157:H7 (Stx2-1717_gp22). With the exception of pMIN27_28 (which contains an in-frame 3 nucleotide insertion relative to other *cII* sequences) all sequences were 297 nucleotides in length and aligned without gaps.

### Statistical analysis

Differences in the level of fluorescence from the PT32 strains compared to PT21/28 strains and strains containing both Stx2 or Stx2c prophages compared to strains only containing one were compared by standard two-sample t-tests on log_10_ transformed values. Standard two-sample t-tests were also performed to compare relative fluorescence in specific TUV, EDL and K-12 strains. A fisher exact test was carried out to compare in PT32 and PT21/28 strains the proportion exhibiting expression levels above a threshold. All analyses were carried out in R (© R Development Core Team (2009). R version 2.10.1(© Foundation for Statistical Computing, Vienna, Austria. ISBN 3-900051-07-0, URL http://www.R-project.org.). P<0.05 was taken to indicate statistical significance. Statistical analysis of qRT-PCR data was carried out using REST 2009 software [69].

## Supporting Information

Figure S1 Analysis of specific transcript levels in the presence and absence of Stx prophages. (A) *ler*, *espD* and *gapA* (control) transcript levels in relation to 16s rRNA from total RNA extracted from TUV93-0 and a derivative containing the Sp5 Stx2 prophage from Sakai, conjugated into TUV93-0 as described in Materials and Methods. (B) *ler*, *espD* and *gapA* (control) transcript levels were determined from EDL933 and derivatives with the Stx1 prophage and both the Stx1 and Stx2 prophages deleted by allelic exchange (table S2). * *p*<0.05, *** *p*<0.001.
(PDF)

Figure S2 The role of *gadE* in Stx phage-based repression of type III secretion. (A) A *gadE*::*gfp* reporter (pP*gadE*.GFP+) and control plasmid (pKC26) (table S2 and [14]) were transformed into *E. coli* K12 and *E. coli* K12 (Sp5) and fluorescence measured throughout the growth curve. Data shown is the mean of three cultures with readings at OD_420 nm_ = 0.9. The presence of the integrated Stx2-prophage (Sp5) led to a significant increase (*p*<0.001) in *gadE* expression. (B) The marked Sp5 prophage was conjugated into TUV93-0 and the *gadE* mutant of this strain (table 1) and the T3S level assessed by Western blotting for EspD. RecA levels were monitored from the pellet as a control. The presence of the integrated Stx2 prophage was able to repress T3S despite the absence of *gadE*, indicating that an increase in *gadE* expression is not responsible for the measured repression as would be anticipated [14].
(PDF)

Table S1 EHEC O157:H7 PT21/28 and PT32 strains and properties.
(PDF)

Table S2 Plasmids used in the study.
(PDF)

Table S3 Oligonucleotide primers used in this study.
(PDF)

Table S4 *cII* homologs found in O157 strains used in this study.
(PDF)
