# Supplementary figures and images for: Lysogeny with Shiga Toxin 2-Encoding Bacteriophages Represses Type III Secretion in Enterohemorrhagic Escherichia coli
Source: PLoS Pathog. 2012 May 17;8(5):e1002672. doi: 10.1371/journal.ppat.1002672 (PMC3355084; doi:10.1371/journal.ppat.1002672)

Figure S1

A

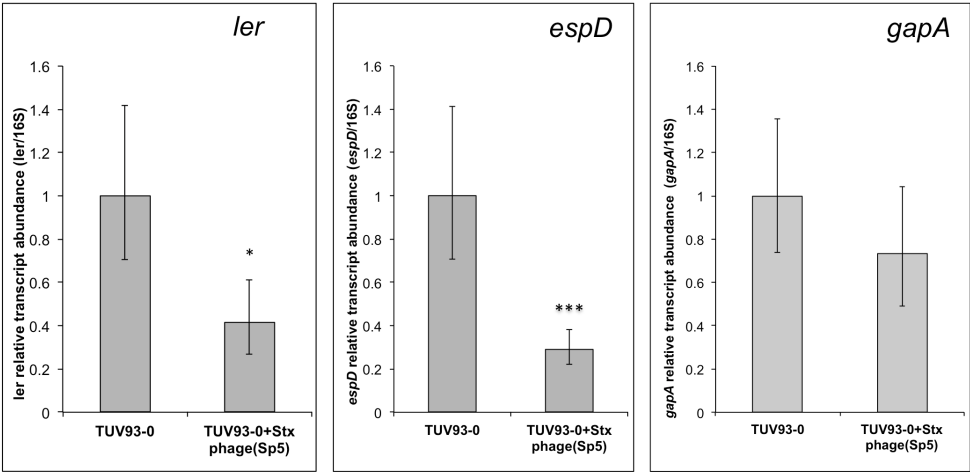

B

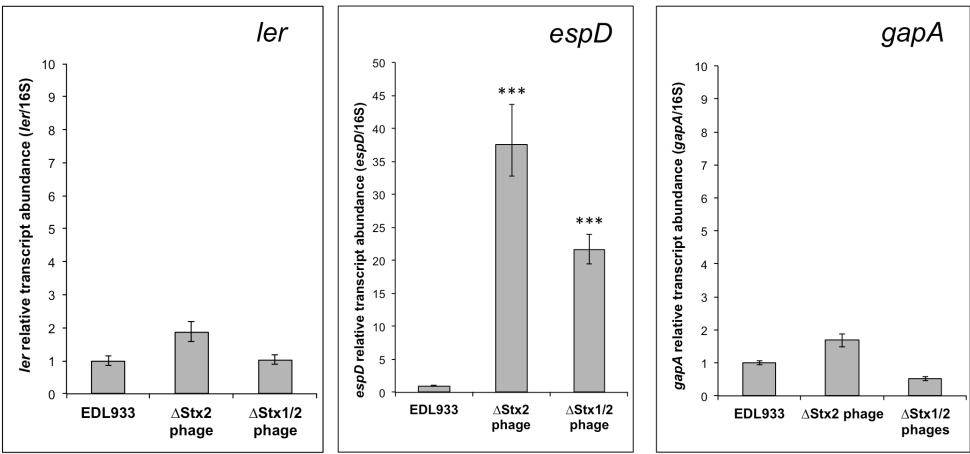

Supplement: Figure S1 — Analysis of specific transcript levels in the presence and absence of Stx prophages. (A) ler, espD and gapA (control) transcript levels in relation to 16s rRNA from total RNA extracted from TUV93-0 and a derivative containing the Sp5 Stx2 prophage from Sakai, conjugated into TUV93-0 as described in Materials and Methods. (B) ler, espD and gapA (control) transcript levels were determined from EDL933 and derivatives with the Stx1 prophage and both the Stx1 and Stx2 prophages deleted by allelic exchange (table S2). * p<0.05, *** p<0.001. (PDF) [file ppat.1002672.s001.pdf]

Figure S2

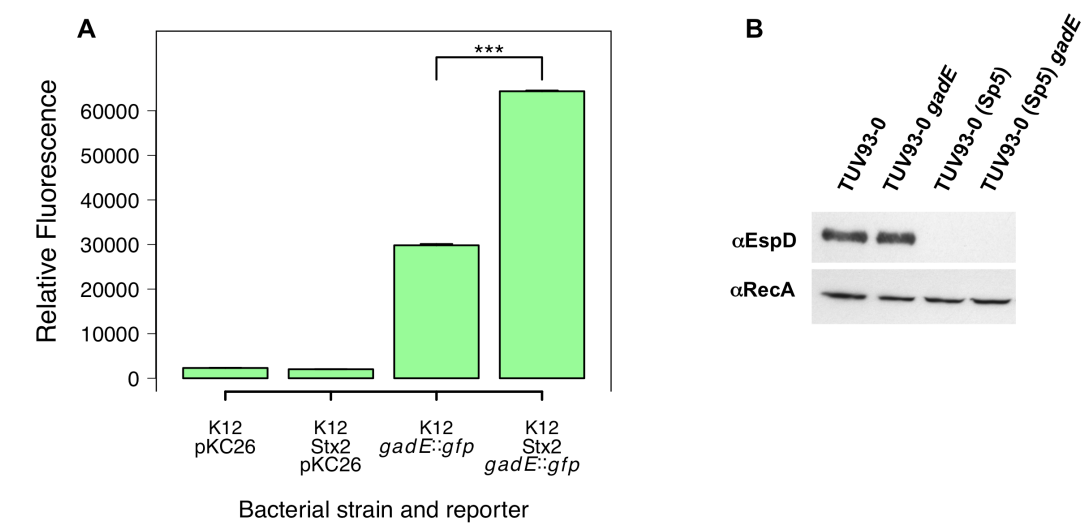

Supplement: Figure S2 — The role of gadE in Stx phage-based repression of type III secretion. (A) A gadE::gfp reporter (pPgadE.GFP+) and control plasmid (pKC26) (table S2 and [14]) were transformed into E. coli K12 and E. coli K12 (Sp5) and fluorescence measured throughout the growth curve. Data shown is the mean of three cultures with readings at OD420 nm = 0.9. The presence of the integrated Stx2-prophage (Sp5) led to a significant increase (p<0.001) in gadE expression. (B) The marked Sp5 prophage was conjugated into TUV93-0 and the gadE mutant of this strain (table 1) and the T3S level assessed by Western blotting for EspD. RecA levels were monitored from the pellet as a control. The presence of the integrated Stx2 prophage was able to repress T3S despite the absence of gadE, indicating that an increase in gadE expression is not responsible for the measured repression as would be anticipated [14]. (PDF) [file ppat.1002672.s002.pdf]
