# Supplementary material for: Lysogeny with Shiga Toxin 2-Encoding Bacteriophages Represses Type III Secretion in Enterohemorrhagic Escherichia coli
Source: PLoS Pathog. 2012 May 17;8(5):e1002672. doi: 10.1371/journal.ppat.1002672 (PMC3355084; doi:10.1371/journal.ppat.1002672)
Supplement: Table S1 — EHEC O157:H7 PT21/28 and PT32 strains and properties. (PDF) [file ppat.1002672.s003.pdf]

Table S1. EHEC O157:H7 PT21/28 and PT32 strains and properties

Source[1]	Original isolate Code[1]	Year isolated	Phage Type[2]	Fecal count (CFU)[2]	Super-shedder >1000 CFU	LEE1-GFP (RFU)[3]	*stx1*	*stx2*	*stx2c*
Bovine	WX006972S01E	2002	21/28	<100	no	7692	-	+	+
Bovine	WX011805S01E	2002	21/28	<100	no	11923	-	+	+
Bovine	WX014281S01E	2003	21/28	<100	no	12692	-	+	+
Bovine	WX015602S01E	2003	21/28	<100	no	15000	-	+	+
Bovine	WX017480S01E	2003	21/28	<100	no	3078	-	+	+
Bovine	WX017845S01E	2003	21/28	300000	yes	5000	-	+	+
Bovine	WX009000S01E	2002	21/28	686400	yes	6154	-	+	+
Bovine	WX017849S01E	2003	21/28	150000	yes	10769	-	+	+
Bovine	WX011806S01E	2002	21/28	384000	yes	12692	-	+	+
Bovine	WX008997S01E	2002	21/28	269200	yes	12692	-	+	+
Bovine	WX017489S01E	2003	21/28	77600	yes	24615	-	+	+
Bovine	WX017706S01E	2003	21/28	16100	yes	NT	-	+	+
Bovine	WX017504S01E	2003	21/28	59300	yes	5385	-	+	+
Bovine	WX017704S01E	2003	21/28	11500	yes	NT	-	+	+
Bovine	WX011820S01E	2002	21/28	42400	yes	10385	-	+	+
Bovine	WX017850S01E	2003	21/28	150000	yes	NT	-	+	-
Bovine	WX017478S01E	2003	21/28	46000	yes	NT	-	+	+
Bovine	WX008998S01E	2002	21/28	21000	yes	NT	-	+	+
Bovine	WX016438S01E	2003	32	36150000	yes	28461	-	+	+
Bovine	WX007666S01E	2002	32	NK	NK	20000	-	-	+
Bovine	WX007784S01E	2002	32	<100	no	20385	-	-	+
Bovine	WX007902S01E	2002	32	<100	no	18846	-	-	+
Bovine	WX008309S01E	2002	32	<100	no	16923	-	-	+
Bovine	WX008801S01E	2002	32	<100	no	16923	-	+	-
Bovine	WX009006S01E	2002	32	900	no	NT	-	-	+
Bovine	WX009064S01E	2002	32	<100	no	14231	-	-	+
Bovine	WX009807S01E	2002	32	<100	no	12692	-	-	+
Bovine	WX010671S01E	2002	32	<100	no	21538	-	-	+
Bovine	WX010693S01E	2002	32	<100	no	NT	-	-	+
Bovine	WX011204S01E	2002	32	<100	no	19230	-	-	+
Bovine	WX014079S01E	2003	32	<100	no	NT	-	+	+
Bovine	WX015000S01E	2003	32	119600	yes	28845	-	+	+
Bovine	WX015289S01E	2003	32	<100	no	13078	-	-	+
Bovine	WX017001S01E	2003	32	<100	no	NT	-	-	+

Bovine	WX017482S01E	2003	32	<100	no	NT	-	+	+
Bovine	WX017742S01E	2003	32	<100	no	13846	-	-	+
Bovine	WX018755S01E	2003	32	<100	no	11923	-	-	+
Bovine	WX020206S01E	2004	32	<100	no	13846	-	-	+
**Source[4]**	**Original isolate code[4]**	**Year isolated**	**Phage Type**	**Fecal count (CFU)**	**Super-shedder >1000 CFU**	**LEE1-GFP (RFU)[3]**	***stx1***	***stx2***	***stx2c***
Human	16370	2008	21/28	-	NA	13461	-	+	+
Human	16308	2008	21/28	-	NA	NT	-	+	+
Human	16117	2008	21/28	-	NA	NT	-	+	+
Human	16000	2008	21/28	-	NA	NT	-	+	-
Human	15919	2008	21/28	-	NA	NT	-	+	+
Human	15827	2008	21/28	-	NA	NT	-	+	-
Human	16347	2008	21/28	-	NA	NT	-	+	+
Human	16299	2008	21/28	-	NA	NT	-	+	+
Human	16094	2008	21/28	-	NA	NT	-	+	+
Human	15999	2008	21/28	-	NA	NT	-	+	+
Human	15960	2008	21/28	-	NA	NT	-	+	+
Human	15883	2008	21/28	-	NA	NT	-	+	+
Human	16161	2008	32	-	NA	36155	-	+	-
Human	15570	2008	32	-	NA	20385	+	-	+
Human	13425	2007	32	-	NA	NT	-	-	+
Human	13024	2007	32	-	NA	NT	-	+	+
Human	12607	2007	32	-	NA	NT	-	+	-
Human	12414	2007	32	-	NA	NT	-	+	+
Human	12443	2007	32	-	NA	NT	-	+	+
Human	16512	2008	32	-	NA	NT	-	+	-
Human	15780	2008	32	-	NA	NT	-	+	+
Human	14052	2008	32	-	NA	NT	-	-	+
Human	13493	2007	32	-	NA	NT	-	+	+
Human	12730	2007	32	-	NA	NT	-	+	-

1. All bovine isolates were from farms in Scotland as described previously [42]. Numbers underlined are the abbreviated designation used to define the isolates when referred to in the manuscript.
2. The phage type and faecal count were determined as described previously [42].
3. LEE1-GFP expression was determined as described in Material and Methods. NT – not tested; NA – not applicable; NK – not known.
4. All human cases were from patient samples collected in Scotland and analysed by the Scottish *E. coli* reference laboratory (SERL).
