# Supplementary material for: Lysogeny with Shiga Toxin 2-Encoding Bacteriophages Represses Type III Secretion in Enterohemorrhagic Escherichia coli
Source: PLoS Pathog. 2012 May 17;8(5):e1002672. doi: 10.1371/journal.ppat.1002672 (PMC3355084; doi:10.1371/journal.ppat.1002672)
Supplement: Table S2 — Plasmids used in the study. (PDF) [file ppat.1002672.s004.pdf]

Table S2. Plasmids used in the study

Plasmid	Details	Source
pAJR32	Source of *sacBkan* cassette	[44]
pBADmyc-HisA/pBAD18	Arabinose inducible expression vectors	Invitrogen
pBR322*Δ**Sca*I-*Ssp*I	pBR322 digested with *Sca*I and *Ssp*I, then ligated. This removes part of the ampicillin resistance gene.	This study
pBAD-N	Amplified with primers JTstx2.N.F.*Nco*I and JTstx2.N.R.*Hind*III from Sakai and cloned via *Nco*I and *Hind*III sites in pBAD18	This study
pBAD-CRO	Amplified with primers JTstx2.CRO.F.*Nco*I and JTstx2.CRO.R.*Hind*III from Sakai and cloned via *Nco*I and *Hind*III sites in pBAD18	This study
pBAD-CI	Amplified with primers JTstx2.CI.F.*Nco*I and JTstx2.CI.R.*Hind*III from Sakai and cloned via *Nco*I and *Hind*III sites in pBAD18	This study
pBAD-CII	Amplified with primers JTstx2.CII.F.*Nco*I and JTstx2.CII.R.*Hind*III from Sakai and cloned via *Nco*I and *Hind*III sites in pBAD18	This study
pBAD-Q	Amplified with primers JTstx2.cI.Q.*Nco*I and JTstx2.Q.R.*Hind*III from Sakai and cloned via *Nco*I and *Hind*III sites in pBAD18	This study
pAJR70	pACYC184 containing *egfp*	[44]
pAJR71	LEE1 promoter fusion	[44]
pIB307	pMAK705-based vector for allelic exchange; temperature-sensitive replicon	[70]
pWSK29	Cloning vector	[Ref 1*]
pXLS01	*ler* with 567 bp of the promoter region was amplified with primer pair Ct-ler-SalI and Nt-ler-PstI, cloned into pWSK29 with *Sal*I and *Pst*I	This study
pXLS10	*z3357* (cII_{stx1}) flanking regions amplified from EDL933 and cloned into pIB307 as described in Material and Methods (used to make ZAP1325)	This study
pXLS11	*z1449* (cII_{stx2}) flanking regions from EDL933 cloned into pIB307 as described in Material and Methods (used to make ZAP1323 & pXLS13)	This study
pXLS12	Stx1 phage (CP-933V) flanking regions from EDL933 cloned into pIB307 as described in Material and Methods (used to make pXLS16)	This study
pXLS13	*z1449* (cII_{stx2}) flanking regions from EDL933 cloned into pIB307 with *sacBkan* cassette (used to make ZAP1321)	This study
pXLS14	*z3357* (cII_{stx1}) flanking regions from EDL933 cloned into pIB307 with *sacBkan* cassette (used to make ZAP1324)	This study
pXLS15	Stx2 phage (BP-933W) flanking regions from EDL933 cloned into pIB307 as described above in Material and Methods (used to make ZAP1322)	This study
pXLS16	Stx1 phage (CP-933V) flanking regions from EDL933 cloned into pIB307 with *sacBkan* cassette (used to make ZAP1326)	This study
pKC26	GFP+ vector for constructing transcriptional fusions	[14]
pP*gadE*.GFP+	GFP+ transcriptional fusion to the *gadE* promoter	[14]

Ref 1* Wang RF, Kushner SR (1991) Construction of versatile low-copy number vectors for cloning, sequencing and gene expression in *Escherichia coli*. Gene 100: 195-9.
