# Supplementary material for: Lysogeny with Shiga Toxin 2-Encoding Bacteriophages Represses Type III Secretion in Enterohemorrhagic Escherichia coli
Source: PLoS Pathog. 2012 May 17;8(5):e1002672. doi: 10.1371/journal.ppat.1002672 (PMC3355084; doi:10.1371/journal.ppat.1002672)
Supplement: Table S3 — Oligonucleotide primers used in this study. (PDF) [file ppat.1002672.s005.pdf]

Table S3. Oligonucleotide primers used in this study.

Primer	Detail	Source
stx2 up 5'	aaaaaGGATCCgacatgttgaaacgatggcac	This study
stx2 up 3'	gggggAAGCTTcgagcaatagacagttcttcc	This study
stx2 down 5'	aaaaaGGATCCcaatatgtccgtacatggaataa	This study
stx2 down 3'	aaaaaGAGCTCgatggctttttgacgagcttaa	This study
stx1 up 5'	aaaaaGGATCCcacgcgcgtaacgtgacag	This study
stx1 up 3'	gggggAAGCTTcgcacgggtattggcaatag	This study
stx1 down 5'	aaaaaGGATCCcgagatcaaacgctggatcg	This study
stx1 down 3'	aaaaaGTCGACgttcgattttcaccgcacca	This study
z1449 up 5'	aaaaaGAGCTCcaaacaacatcaacgtttagaa	This study
z1449 up 3'	aaaaaGGATCCttcttaagatttccaatag tgaa	This study
z1449 down 5'	aaaaaGGATCCgggaattactggatcaatcc	This study
z1449 down 3'	cccccAAGCTTgaaagatcgttttctggctg	This study
z3357 up 5'	aaaaaGTCGACctttcttgttcctaactcgtag	This study
z3357 up 3'	aaaaaGGATCCttgttaattttcctatattgatattg	This study
z3357 down 5'	aaaaaGGATCCgggaattactggatcaatccac	This study
z3357 down 3'	gggggAAGCTTgtcgttttctggctggtcaga	This study
yehU	cacatctgctgaagcagcagc	This study
Z3305	gcgatacagatctcaacacgc	This study
intV	atcacgaacagatagaactcc	This study
yehV	agcgtctgggcggggtaatcc	This study
wrbA	cgcccgtctggtcgaggaagg	This study
intW	tacaatgtttatggtgcatgg	This study
z1503	tttttccctcgcccataacc	This study
z1504	cggaatagagataacacgagg	This study
SLT-I-1	cagttaatgtggtggcgaag	[Ref 1*]
SI-1	cgcctgctattttcactgagc	[Ref 1*]
z1449 5'	gctattttcacaatggacattc	This study
z1449 3'	tcagaattgcatatcaatttgc	This study
z1449 external 5'	cacgcatacctttcaactagc	This study
z1449 external 3'	ctgtggattgatccagtaattc	This study
z3357 5'	aaaaaGAATTCtcagaattgcatatcaatttgc	This study
z3357 3'	aaaaaCCATGGatggcacaagcaagctacag	This study
z3357 external 5'	gtcataatgaatcctgtggatt	This study
z3357 external 3'	ggtgtgccgctataactcaa	This study
SacB 5'	gcaactcaagcgtttgcgaaag	This study
SacB 3'	ggcttgtatgggccagttaaag	This study
stx2 5'	gcggttttatttgcattagc	[64]
stx2 3'	tcccgtcaaccttcactgta	[64]
stx2c 5'	gcggttttatttgcattagt	[64]
stx2c 3'	agtactcttttccggccact	[64]
Ct-ler-SalI	aaaaaGTCGACtcatgttaaatatttttcagcgg	This study
Nt-ler-PstI	aaaaaCTGCAGgtatcatatagcatcatatagtg	This study
JTstx2.N.F.*Nco*I	ggCCATGGcacgcagaactcagtt	This study
JTstx2.N.R.*Hind*III	ggAAGCTTtcacctcgccgtcagttgtt	This study
JTstx2.CRO.F.*Nco*I	ggCCATGGaaaatcttgatgagcc	This study
JTstx2.CRO.R.*Hind*III	ggAAGCTTtatgcagccagaaggttct	This study
JTstx2.CI.F.*Nco*I	ggCCATGGttcagaatgaaaaagt	This study
JTstx2.CI.R.*Hind*III	ggAAGCTTtcacgaacttttcagccact	This study
JTstx2.CII.F.*Nco*I	ggCCATGGaacaaacaagttacag	This study
JTstx2.CII.R.*Hind*III	ggAAGCTTtcagaattgcatatcaattt	This study
JTstx2.Q.F.*Nco*I	ggCCATGGgtgatatccggcaggttc	This study

Primer	Detail	Source
JTstx2.Q.R.*Hind*III	ggAAGCTTttacgatcgtaaactatttttcg	This study
CIseq1	gatggttccagtacagagc	This study
CIseq2	ccgcttgatactcatggcc	This study
RT-ler.F	ctgcgagagcaggaagttca	[15]
RT-ler.R	aggcacattagtatacccagctc	[15]
RT-16S.F	attgacgttacccgcagaag	[15]
RT-16S.R	cgctttacgcccagtaattc	[15]
RT-espD.F	cgcacaagctatccctatctc	This study
RT-lespD.R	actttctgcgcggaagtatc	This study
RT-gapA.F	gggacgaagttggtgttgac	[14]
RT-gapA.R	aaccactttcttcgcaccag	[14]

Ref 1* Olsvik O and Strockbine NA (1993) PCR detection of heat-stable, heat labile and Shiga-like toxin genes in *Escherichia coli*. Diagnostic molecular microbiology. Washington, DC: American Society for Microbiology. pp. 271-276.
