# Supplementary material for: Lysogeny with Shiga Toxin 2-Encoding Bacteriophages Represses Type III Secretion in Enterohemorrhagic Escherichia coli
Source: PLoS Pathog. 2012 May 17;8(5):e1002672. doi: 10.1371/journal.ppat.1002672 (PMC3355084; doi:10.1371/journal.ppat.1002672)
Supplement: Table S4 — cII homologs found in O157 strains used in this study. (PDF) [file ppat.1002672.s006.pdf]

Table S4. *cII* homologs found in O157 strains used in this study

Strain	Shiga toxin	Locus_tag	Phage	Accession	Nucleotide Identity[1]	Amino acid Identity[1]
EDL933	Stx2	Z1449	BP-933W	gi|12514302	100%	100%
EDL933	Stx1	Z3357	CP-933V	gi|12516410	94.6%	94.9%
EDL933	N/A	Z0310	CP-933H	gi|12513024	56.5%[2]	28.3%[2]
Sakai	Stx2	ECs1187	Sp5	gi|13360647	96.0%	95.9%
Sakai	Stx1	ECs2988	Sp15	gi|13362457	94.6%	94.9%
Sakai	N/A	ECs0276	Sp1	gi|13359733	56.5%[2]	28.3%[2]

[1] Identities were calculated from pair-wise alignments with z1449, the EDL933 Stx2 *cII* sequence.
[2] Z0310 and ECs0276 are identical sequences detected in a BLASTp search using z1449 as the query sequence against a database of all Sakai or EDL933 protein coding sequences (E-value = 5e-04).
